# Evaluation of the Amharic version of the London measure of unplanned pregnancy in Ethiopia

**DOI:** 10.1371/journal.pone.0269781

**Published:** 2022-06-13

**Authors:** Ararso Baru Olani, Tariku Bekelcho, Asfawosen Woldemeskel, Kibreyesus Tefera, Degefe Eyob

**Affiliations:** 1 College of Medicine and Health Sciences, Arbaminch University, Arbaminch, Ethiopia; 2 Research and Collaboration Department, Slum and Rural Health Initiative Network, Addis Ababa, Ethiopia; 3 Department of Medicine, College of Health Sciences, Ethiopian Police University, Sendafa, Ethiopia; University of Salamanca, SPAIN

## Abstract

**Background:**

Unplanned pregnancy is an important public health problem in both the developing and developed world, as it may cause adverse social and health outcomes for mothers, children, and families as a whole. London Measure of Unplanned Pregnancy (LMUP) has been formally and informally validated in multiple and diverse settings. However, there is a dearth of literature on the validation of LMUP in Ethiopia either in the Amharic version or other languages.

**Objective:**

The general objective of this study was to translate the LMUP into Amharic and evaluate its psychometric properties in a sample of Amharic-speaking women receiving antenatal care (ANC) service at public health facilities in Arbaminch and Birbir towns.

**Methods:**

A cross-sectional study design was used for the study. Forward and backward translation of original English LMUP to Amharic was done. A cognitive interview using a pretested structured questionnaire was used to collect the data from respondents. The collected data was analyzed using SPSS version 25. Reliability was assessed using Cronbach’s alpha, inter-item correlations, and corrected item-total correlations while construct validity was assessed using principal components analysis and hypothesis testing.

**Results:**

Data was collected from 320 women attending antennal care services at selected public health care facilities. LMUP range of 1to 11 was captured. The prevalence of unplanned pregnancies was 19(5.9%), while 136(42.5 were ambivalent and 165(51.6%) were planned pregnancies. The reliability testing demonstrated acceptable internal consistency (Cronbach’s alpha = 0.799) and the validity testing confirmed the unidimensional structure of the scale. In addition, all hypotheses were confirmed.

**Conclusions:**

Amharic version of LMUP is a valid and reliable tool to measure pregnancy intention so that it can be used by Amharic speaking population in Ethiopia. It can also be used in research studies among Amharic-speaking women to measure unplanned pregnancy.

## Background

Unintended pregnancy is a pregnancy that has been reported to have been either unwanted (occurred when no child or children are desired) or mistimed (occurred earlier than desired) [1]. It is an important public health concern in the world, because of its association with adverse social and health outcomes for mothers, children, and the community as a whole [2–4].

Unintended pregnancy accounted for approximately 121 million pregnancies each year between 2015 to 2019 [5]. Developing countries are contributing a substantially higher number of unintended pregnancies than developed countries [5, 6]. Between 2015 to 2019, on average, the annual rate of unintended pregnancies in sub-Saharan African countries was 91 pregnancies per 1000 women aged 15–49 years [5]. In Ethiopia, a finding from a systematic review reported a 28% prevalence of unintended pregnancy in the country [7]. The prevalence of unintended pregnancy in Ethiopia according to 2016 Ethiopian Demographic and Health Survey data (EDHS) was 26.6% [8]. Another Ethiopian study reported the prevalence of unintended pregnancy as high as 41.5% [9].

Even though the global unintended pregnancy rate is recently declining, the proportion of unintended pregnancies ending in abortion is increasing worldwide in general and in developing countries in particular [5, 6]. Globally, it was estimated that about 61% of all unintended pregnancies, ended in induced abortion [5]. Global estimates from 2010 to 2014 showed that about 45% of all abortions were carried out under unsafe conditions and almost all of them took place in developing countries [10]. It was reported that unsafe abortion is attributed to about 4.7% to 13.2% of global maternal deaths every year [11]. Although abortion law is semi-liberal in Ethiopia [12], 47% of all abortions still occurred outside of health facilities in the country [13].

In addition to the risk associated with induced abortions, unintended pregnancy exposes women to delayed antenatal care visits, increased risks of obstetric complications, increased risks of perinatal depression, unnecessary financial expenditure, and reduced educational opportunities [14–16]. Moreover, children born of unplanned pregnancies have been shown to have a lower birth weight, stunted growth, poor mental and physical health during childhood, and are less likely fully vaccinated compared to other children [2, 17].

Most of the available studies in Ethiopia measured pregnancy intention by asking women a single question regarding whether their pregnancy is entirely unwanted or wanted but at a later time [8, 9, 18–20]. However, it has been realized that the concept of unintended pregnancy is potentially more complex and requires continuous or multi-item measurement strategies than asking a single question [1, 21, 22].

To address the complexity of measuring pregnancy intention, a psychometrically valid measure of pregnancy intention known as the London Measure of Unplanned Pregnancy (LMUP) was developed [23]. LMUP measures pregnancy intention on a six-item scale and it was developed in the United Kingdom [23]. Since its development, translated and culturally adapted versions of the tool was validated in various countries across the world [24–32]. The tool’s simplicity and reliability led to its widespread application to measure unintended pregnancy in different parts of the world [31, 33, 34].

Despite the high prevalence of unintended pregnancy and an increasing trend of induced abortion in Ethiopia [7, 13], to our knowledge, there is no uniform method and validated tools to measure pregnancy intention in the country. Therefore, this study was aimed at evaluating the psychometric properties of the Amharic version of LMUP among Amharic-speaking pregnant women receiving antenatal care services at public health care facilities (one hospital and health centers) in Arba Minch and Birbir towns, Ethiopia.

## Methods

### Study area and study period

The study was conducted on women attending antenatal care follow-ups at public health care facilities found in Arba Minch and Birbir towns. Arba Minch town is the administrative capital of the Gamo zone, which is found in the Southern Nations Nationalities and People’s Regional State (SNNPR) of Ethiopia. The city is found at 505 km toward the South of Addis Ababa, the capital of Ethiopia. The Arba Minch city was founded in 1962 when the capital of the Gamo zone was transferred from Chencha to Arba Minch town and since then, has served as the capital of the Gamo zone. It is the largest town in the Gamo zone and the second town in SNNPR next to Hawassa (39). Similarly, Birbir town is located in the Gamo zone at 465 km toward the South of Addis Ababa. Arba Minch town has three health centers and one general hospital whereas Birbir town has one health center. This study purposively included three health centers and one hospital namely Sikela health center, Secha health center, Birbir health center, and Arba Minch general hospital. Sikela and Secha health centers are found in Arbaminch town while Birbir health center is found in Birbir town. The study was conducted from November 20 to December 22, 2020.

### Study design

An institutional-based cross-sectional study design was carried out to evaluate the psychometric properties of Amharic LMUP among pregnant women attending ANC services.

### Source and study populations

The source populations for this study were all Amharic-speaking women attending ANC services in Arbaminch and Birbir towns. Whereas, the study population was all Amharic-speaking antenatal women who attended ANC services at selected public health care facilities in the study area during the study period.

### Inclusion criteria

All pregnant women attending ANC follow-ups at selected public health facilities from November to December 2020.

### Exclusion criteria

A pregnant woman who was unable to speak Amharic did not volunteer to participate in the study, was critically ill, or attended ANC follow-up at selected facilities for the second or more time while the study was ongoing was excluded from the study.

### Sample size determination

The sample size was estimated using single population proportion formula with the following assumptions: taking the 28% prevalence of unintended pregnancy in Ethiopia from the previous study [7], setting the level of confidence (α) at 0.05 (Z (1-α) = 1.96), and assuming 5% margin of error to the study. Considering a 10% for non-response rate, a 341 sample size was estimated for the study.

### Data collection instrument and procedures

LMUP has six questions and each question scored 0,1 and 2. The maximum value of LMUP gives 12 while the minimum value is 0. The lower the score, the higher tendency of unplanned pregnancy. The score cut-point of less than 3 indicates unplanned pregnancy, the scores of 4 to 9 indicate ambivalence of planning pregnancy while more than 10 indicates planned pregnancy [35].

The translation of LMUP followed the WHO’s recommendation on research instrument translation and adaptation [36]. Accordingly, the forward translation of the English LMUP was independently done by two Amharic native speakers. Both of them were lecturers at Arbaminch University and were aware of the purpose of LMUP. The Malawi version of the LMUP was used as the starting point of the forward translation [24]. The expert translation was done by two researchers who have a background in reproductive health and are fluent in Amharic. Backward translation was done by a language professional who has a Master of Arts in English Language and literature.

The original LMUP was initially designed to be filled by the respondents [23]. However, due to the low literacy rate of respondents in the study area, LMUP was adapted for interviewer administration similar to previous studies from Malawi and Brazil [26, 37]. In addition, question six was adapted to the local context using previous experience in Malawi [35].

### Data quality assurance

The training was given to data collectors for common understanding. A pretest was done on seventeen antenatal women receiving antennal care services at Arba Minch General Hospital using the cognitive interview technique. A revision was made to the tool based on the feedback from the pretest, particularly related to the wording of the items. A systematically structured questionnaire was used to collect data and the completeness of the data was checked after completing each questionnaire to maintain data quality.

### Data analysis and presentation

The collected data were analyzed using SPSS version 25. Frequency and percentages were used to summarize the findings while tables and graphs were used to present the data.

The reliability of the study was evaluated using Cronbach’s α statistic with the standard cut-off point value of 0.7. In addition, inter-item correlations were evaluated to check internal consistency. Construct validity was examined using principal components analysis and hypothesis testing. The hypothesis used to examine structural validity was adapted from previous studies that validated LMUP in various languages and cultures and contextualized to the local culture of the study setting [25–28, 35]. Since our data distribution was not normal, non-parametric tests (Mann-Whitney and Kruskal-Wallis tests) were used to test the hypothesis.

### Ethics approval and consent to participate

Ethical approval was obtained from the Ethical Review Board of Arbaminch University, College of Medicine and Health Sciences. A formal support letter was written to each facility by a school of nursing to facilitate the data collection process. The purpose, general content, and nature of the study were explained in the language preferred by each respondent. Verbal consent was obtained from each participant due to the low literacy rate of respondents and the procedure was approved by IRB. The participants’ consent was documented using audio records. The respondents were informed that they had the right to be involved or refuse to participate in the study. Additionally, the respondent had the right to withdraw from the study at any time during the interview. The participants were assured that the data would be handled exclusively by the investigators and no one would be able to recognize them in the report. The confidentiality of the information obtained from each participant was maintained. Furthermore, all procedures involved in this study have adhered to the principles of the Helsinki Declaration.

## Results

### Socio-demographic characteristics

A total of 320 women have included in the study, however, giving a response rate of 91.7%. Among 320 pregnant women attending ANC follow up at the selected facilities, nearly half of the study participants (46.9%) belonged to the age groups of 15 to 24 years, and 35% were found in the age group of 25 to 34 years.

Two-hundred and forty (75%) respondents were urban residents while 80(25%) were rural inhabitants. Most of the study participants were married 306 (95.6%). Concerning employment status, more than one-third of the respondents were housewives 117 (36.6%) followed by civil servants 86(26.9%). Of the total study participants, 290(90.6%) were living with their husbands or partner while 30(9.4%) were living with their families (Table 1).

**Table 1:** Baseline characteristics of the pregnant women seeking antenatal service at public health care facilities in Arbaminch and Birbir towns, Ethiopia.

Variables	Frequency (n = 320)	Percentage (%)
**Age group**		
**15–24**	150	46.9
**25–34**	112	35.0
**35–45**	58	18.1
**Place of residence**		
**Urban**	240	75.0
**Rural**	80	25.0
**Educational status**		
**Unable to read and write**	11	3.4
**Can read and write**	38	11.9
**Completed primary school**	65	20.3
**Completed secondary school**	84	26.3
**Diploma**	71	22.2
**Bachelor**	41	12.8
**Postgraduate**	10	3.1
**Marital status**		
**Married**	306	95.6
**Unmarried**	14	4.4
**Occupation**		
**Housewife**	117	36.6
**Government employee**	86	26.9
**Employed by a private company**	43	13.4
**Merchant**	48	15.0
**Unemployed**	4	1.3
**Others**	22	6.9
**Living arrangement**		
**Husband/partner**	290	90.6
**Family**	30	9.4
**Gravida**		
**Primigravida**	71	22.2
**Multigravida**	249	77.8
**Parity**		
**Para 0**	71	22.2
**Para 1**	84	26.3
**Para 2**	85	26.6
**Para 3 and above**	80	25.0
**Number of alive children**		
**No child**	71	22.2
**One child**	90	28.1
**Two or more child**	159	49.7
**Experienced any form of IPV**		
**Yes**	14	4.4
**No**	306	95.6

Seventy-one (22.2%) respondents were primigravida, and 249(77.8%) were multigravida. Concerning parity, over one-fourth (26.3%) of the study participants were para zero and about 27% were para one (26.6%). Among the total respondents, 71(22.2%) had no child, 90(28.1%) had one child and 159(49.7%) had two or more children. Nearly 5% of the study participants had experienced intimate partner violence during the current pregnancy (Table 1).

### Distribution of Amharic LMUP

**Fig 1:**
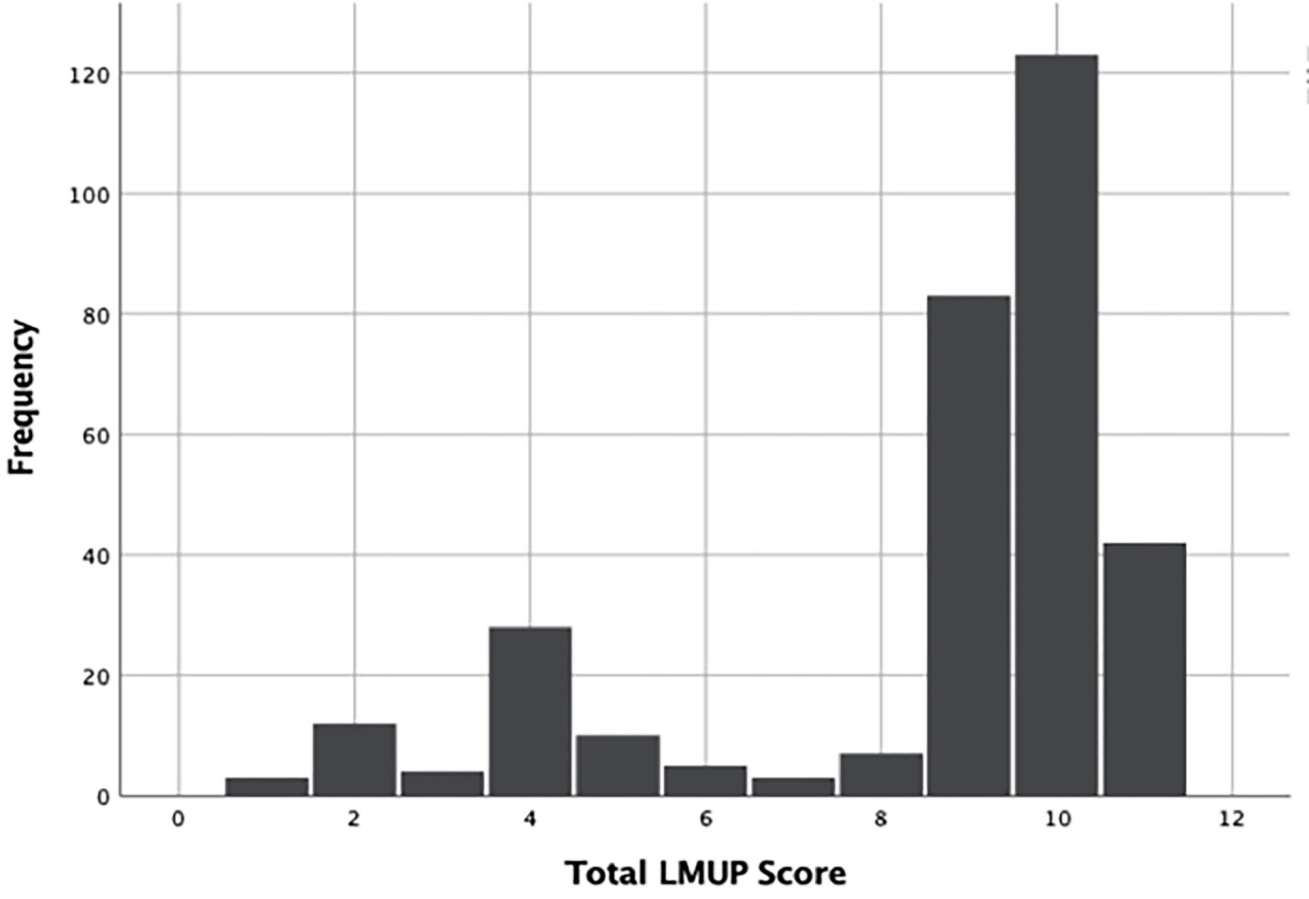
Distribution of Amharic London Measure of Unplanned Pregnancy (LMUP) score.

The distribution of Amharic LMUP was not normal (Skewed to the left). In addition, the full range of the LMUP score was not captured (Fig 1).

### Endorsement frequency of Amharic LMUP

Missing data were not observed for each item. Two of the questions had a response option with more than 80% endorsement. These items were item pregnancy timing and item desire for the baby (Table 2).

**Table 2:** Endorsement frequencies of LMUP items and response options.

Item	Category	Frequency	Percentage
**Use of contraception**	0. Always using contraception	93	29.1
	1. Using contraceptives but not on every occasion	155	48.4
	2. not using contraception	72	22.5
**Timing of pregnancy**	0. Wrong time	24	7.5
	1. Ok but not quite the right time	31	9.7
	2. Right time	265	82.8
**Pregnancy intention**	0. Didn’t intend to get pregnant	43	13.4
	1. Intentions kept changing	23	7.2
	2. Intended to get pregnant	254	79.4
**The desire for a baby**	0. Didn’t want a baby	18	5.6
	1. Mixed feelings about having a baby	40	12.5
	2. Wanted baby	262	81.9
**Partner discussion**	0. Never discussed getting pregnancy	24	7.5
	1. Discussed but didn’t agree to get pregnant	43	13.4
	2. Agreed to get pregnant	253	79.1
**Preparation for pregnancy**	0. No preparatory lifestyle changes	78	24.4
	1. Did one preparatory lifestyle changes	241	75.3
	2. Did two or more preparatory lifestyle changes	1	0.3

### Categorization of LMUP score

The majority of the respondents had planned pregnancy followed by ambivalence in the intention of the current pregnancy, which accounts for 51.6% and 42.5% respectively (Table 3).

**Table 3:** Distribution of LMUP score by category.

Category	LMUP Score	Frequency(n = 320)	Percentage
**Planned**	10–12	165	51.6
**Ambivalent**	4–9	136	42.5
**Unplanned**	0–3	19	5.9

### Reliability of Amharic LMUP

The result for Cronbach’s alpha of the whole scale was 0.799, which indicated acceptable internal consistency (Table 4). In addition, an inter-item correlation matrix was done to further evaluate the reliability of the Amharic LMUP. Accordingly, all inter-item correlations were positive except for the contraceptive use before the current pregnancy (Table 5). Furthermore, corrected item-total correlations were >0.2 for all items except for item 1 (contraception use) (Table 6).

**Table 4:** Reliability statistics of Amharic version of LMUP.

Reliability Statistics	
**Cronbach’s Alpha**	N of Items
**.799**	6

**Table 5:** Inter-item correlation matrix of Amharic version LMUP.

Inter-item correlation matrix							
S.N		Use of contraception	Timing of pregnancy	Pregnancy intention	The desire for a baby	Partner discussion	Preparation for pregnancy
**1**	Use of contraception	1.000	-.243	-.157	-.129	-.095	-.171
**2**	Timing of pregnancy	-.243	1.000	.699	.669	.703	.657
**3**	Pregnancy intention	-.157	.699	1.000	.888	.876	.755
**4**	The desire for a baby	-.129	.669	.888	1.000	.896	.713
**5**	Partner discussion	-.095	.703	.876	.896	1.000	.763
**6**	Preparation for pregnancy	-.171	.657	.755	.713	.763	1.000

**Table 6:** Corrected item-total correlation with Cronbach’s alpha.

Items	Corrected item-total correlation	Cronbach’s alpha if an item deleted
**Use of contraception**	-0.175	0.937
**Timing of pregnancy**	0.647	0.747
**Pregnancy intention**	0.840	0.687
**The desire for a baby**	0.849	0.704
**Partner discussion**	0.880	0.688
**Preparation for pregnancy**	0.735	0.744

### Validity of the Amharic LMUP

#### Structural validity

Kaiser-Meyer-Olkin Measure (KMO) and Bartlett’s Test was done to check whether our data were suitable for factor analysis. Our finding revealed that the data is suitable for factor analysis because we obtained the KMO value of 0.880, and Bartlett’s for sphericity was statistically significant with a p-value of < 0.0001(viz. p = 0.000) (Table 7).

**Table 7:** Kaiser-Meyer-Olkin Measure of Amharic LMUP.

KMO and Bartlett’s Test		
**Kaiser-Meyer-Olkin Measure of Sampling Adequacy.**		.880
**Bartlett’s Test of Sphericity**	Approx. Chi-Square	1636.072
	Df	15
	Sig.	.000

#### Principal component analysis

**Fig 2:**
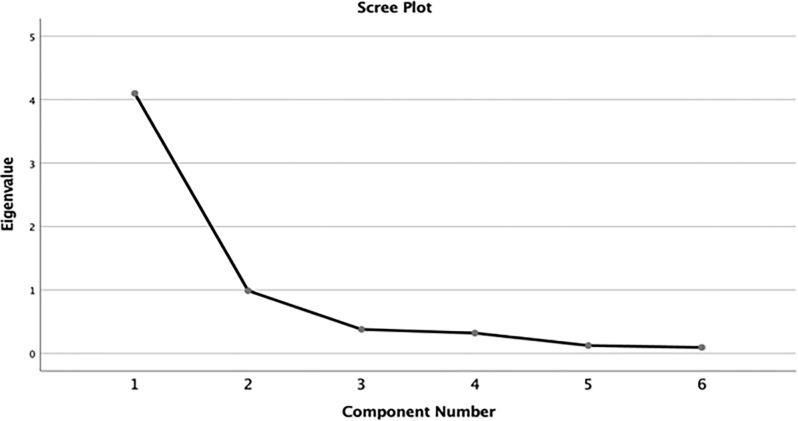
Scree plot of Eigenvalues for Amharic LMUP.

Generally, Eigenvalues have to be greater than or equal to one. Our findings showed an Eigenvalue greater than 1 for one of the items (item 1 with an Eigenvalue of 4.098), which is very important (Table 8 and Fig 2). The total variance explained was 68.3% (Table 8).

**Table 8:** Principal component analysis and component loadings.

Component	Eigenvalues		Component Loadings (Eigenvalue 4.098 for component 1)
	Total	% of Variance	
**1**	4.098	68.303	-2.227
**2**	.987	16.448	0.826
**3**	.378	6.300	0.939
**4**	.320	5.329	0.927
**5**	.123	2.057	0.940
**6**	.094	1.563	0.861

*Construct validity hypothesis*. Table 9 shows that all hypotheses tested confirmed the construct validity of the Amharic version of LMUP.

**Table 9:** Construct validity hypothesis tests.

Hypothesis	Variables	Score range (median)	p-value
**The youngest women will have the lowest scores**	Age group		0.031*
	15–24	1-11(10)	
	25–34	1-11(9)	
	35–45	2-11(10)	
**Women living in the rural area will have the lowest score**	Place of residence		0.052*
	Urban	1-11(10)	
	Rural	2-11(9)	
**Unmarried women will have the lowest score**	Marital status		<0.001**
	Married	1-11(10)	
	Unmarried	2-10(4)	
**Women living with their husbands or partner will have the highest score**	Living arrangement		<0.001**
	Husband/partner	10(1–11)	
	Family	4(1–11)	
**Nulliparous women will have the highest score**	Parity		
	Nulliparous	1-11(10)	
	Para 1	2-10(9)	
	Para 2	1-11(9)	
	Para 3 and above	2-11(10)	
**IPV is associated with the lower score**	Experienced any form of IPV		0.029*
	Yes	2-10(8)	
	No	1-11(10)	

* P-value obtained via Mann-Whitney test

** P-value obtained via Kruskal-Wallis test

## Discussion

The objective of this study was to translate the London Measure of Unplanned Pregnancy (LMUP) into Amharic and evaluate its psychometric properties in a sample of Amharic-speaking women receiving ANC services at selected public health care facilities in Ethiopia.

The importance of understanding women’s pregnancy planning and intentions for preconception care and engagement were reported by scholars as crucial for pregnancy planning [28, 38]. The proportion of planned pregnancy was 51.6% in the present study which was far lower than that of findings of the Dutch version from Belgium and the English version from Australia, which were reported as 84.7% and 74.4% respectively [27, 38]. This disparity could be attributable to the fact that the study population in the present study was heterogeneous in terms of age of first marriage in which the majority of the women (46.9%) belonged to the 15–24 age group which could make them unfamiliar with pregnancy planning. Lower literacy rates and being housewives and unemployed could also make them economically dependent on their partner and probably predispose them to the difficulty of having planned pregnancy.

Results of the current study provided evidence of validity and reliability of the Amharic LMUP which provided evidence of technical validity of the Amharic version of LMUP, in so doing confirming its content validity which is suitable to be tailored in our context and as compared with the Arabic version LMUP [28].

Concerning the score distribution of LMUP in the present study, it was left-skewed which was comparable with the result reported in the studies conducted in Belgium and Australia [27, 38]. This similarity could be explained by the fact that there was access to antenatal care-related information from health extension workers which resulted in a good response rate for questions related to pregnancy planning, desire, and other components.

Our study findings confirmed the reliability of Amharic LMUP with Cronbach’s α of 0.799 and inter-item correlations, which were all positive except for the use of contraception. Our study findings were comparable with the Australian study, the Chichewa version of LMUP from Malawi, the Dutch version from Belgium, and the Arabic LMUP version from Saudi Arabia [27, 28, 37, 39].

Evidence of structural validity of items of the Amharic version of LMUP was established with a KMO value of 0.880, and Bartlett’s for sphericity with a statistically significant p-value of < 0.0001. This confirmed suitability of our data for factor analysis. The present finding was comparable with a study done in Saudi Arabia, which reported a KMO test value of 0.885 and Bartlett’s for Sphericity with a statistically significant p-value of <0.0001 [28].

Our results of the component analysis confirmed the unidimensional structure of the scale, with one component Eigenvalue of 4.098 for which all variables were loaded onto one component to measure the same construct. In addition, the factor matrix confirmed the items’ correlation with the measured scale implying that there was a strong correlation between pregnancy intention and pregnancy desire, desire for the child, and discussion with a partner. This confirmed the validity of the Amharic version of LMUP as a measure of pregnancy intention. The present findings were in agreement with the original UK study and other translated versions of LMUP from Malawi, Belgium, and Saudi Arabia [23, 27, 28, 39]. Conversely, item one (contraceptive use) was negatively correlated with all other items in the measure which is an unusual finding when compared with the findings of the previous studies [30, 31]. Based on the finding, the authors looked at the data set if there was any coding error with item one and concluded that there was no error in the coding. The reason for the negative correlation could be that item one has not been understood by women in our study. Therefore, further studies should evaluate the Amharic version of item one.

All hypotheses tested in the present study further confirmed the structural validity of the Amharic LMUP, which was comparable to the original LMUP in the UK [23] and other subsequent translated versions of studies in Belgium of the Dutch version [27] and Saudi Arabia of the Arabic version [28].

### Limitations

This study was not free of limitations. First, the original LMUP was designed for self-completion by respondents. However, in the present study, we used interviewer-administered techniques to collect the data due to the lower literacy rate of our respondents. This might lead the respondents to desirability bias as interviewer-administered questionnaires may limit participants’ freedom to share their pregnancy planning behavior and share sensitive information about the construct. Second, as the present study was an institutional-based study the women who were not attended antenatal care were not included in the study and this could limit the generalizability of our findings.

Being the first study to validate and utilize a tool that measures pregnancy planning in Amharic, the use of a multicenter study, and inclusiveness of women from urban and rural communities was among the most significant strength of our study.

## Conclusions

In conclusion, the findings of this study generated evidence that the Amharic version of LMUP is a valid and reliable tool to measure pregnancy intention among Amharic-speaking women. Our findings indicate that the Amharic version of LMUP can be used to measure pregnancy intentions among Amharic-speaking women. Therefore, implementation of this measurement tool in all maternity healthcare, research and policy settings can provide a relatively accurate level of pregnancy intention among Amharic speaking population in Ethiopia. This study could also be used as starting point for further studies to be conducted to validate LMUP in others Ethiopian languages.

## Supporting information

S1 File (SAV)

## Abbreviations

ABO: Ararso Baru Olani
AGH: Arbaminch General Hospital
ANC: Antenatal Care
EDHS: Ethiopian Demographic Health Survey
FP: Family Planning
KMO: Kaiser-Meyer-Olkin Measure
LMUP: London Measure of Unplanned Pregnancy
SNNPR: Southern Nation Nationalities and Peoples Region
